# Sustainable Bio-Based Plasticizers: Advances in Polyols and Natural Compound Derivatives from Sorbitol, Glycerol, Cardanol, and Limonene

**DOI:** 10.3390/polym18080985

**Published:** 2026-04-18

**Authors:** Asma M. Ghazzy, Ala’a S. Shraim, Tabarak R. Al-Sammarraie, Wurood M. Al-Mohammadi, Afnan H. Al-Hunaiti

**Affiliations:** 1 Faculty of Pharmacy, Al-Ahliyya Amman University, Amman 19111, Jordan; wuroodalmahmood843@gmail.com; 2 Department of Medical Laboratory Sciences, Allied Medical Sciences, Al-Ahliyya Amman University, Amman 19111, Jordan; a.shraim@ammanu.edu.jo; 3 Department of Pharmacy, Al-Farahidi University, Baghdad 00964, Iraq; tabarak.riyadh@uoalfarahidi.edu.iq; 4 Department of Chemistry, University of Jordan, Amman 11942, Jordan; a.alhunaiti@ju.edu.jo

**Keywords:** bio-based plasticizers, sustainable plasticizers, polyol ester plasticizers, sorbitol-based plasticizers, glycerol-derived plasticizers, cardanol-based plasticizers, limonene-based plasticizers, phthalate-free polymer additives, polyvinyl chloride (PVC), polylactic acid (PLA)

## Abstract

The rapidly growing concern over the hazardous impact of phthalates on the environment and public health has led to a critical need for alternative and environmentally friendly plastics. Plasticizers developed from natural materials represent one possible solution. This paper explores four types of renewable feedstocks (sorbitol/polyols, glycerin, cardanol from cashew nutshell liquid, and limonene from citrus peels) as sources for developing alternative plasticizer systems. Key areas explored include the type of feedstock utilized, the methods used for extracting or processing the feedstocks, the nature of the chemical modification processes (e.g., esterification, epoxidation, etherification, or reactive grafting) applied to generate the respective plasticizers, and the resultant physical and mechanical properties. The performance of each plasticizer system in polymers such as PVC, PLA, and polysaccharide-based bioplastics is evaluated, alongside the compatibility with biological tissues, toxicological properties, biodegradability, and chemical migration into food simulants. The feasibility of each family of plasticizers is also assessed from an economic perspective, including availability of the feedstocks, economies of scale associated with large-volume production, and competitive pricing relative to established petroleum-derived plasticizers. Overall, sorbitol/polyol and glycerin derivative families have reached a level of maturity that provides a good balance of processability, food-contact safety, and biodegradability. Cardanol-based systems provide an attractive option where aromatic functional groups and combined plasticization–stabilization effects are needed. Limonene-derived plasticizer systems appear promising for use in PLA, but their broader utility may be limited by volatility, strong odors, and susceptibility to oxidation. Common issues identified across all four families include chemical migration into food products, regulatory approval, and the need for detailed life-cycle assessments.

## 1. Introduction

Plasticizers are indispensable additives in contemporary polymer science: they impart flexibility and processability to otherwise brittle polymer matrices such as polyvinyl chloride (PVC), polylactic acid (PLA), and polysaccharides. Phthalates have dominated the plasticizer market for decades owing to their low cost and high effectiveness. However, the widespread presence of phthalates in the environment and in human biological samples has raised serious public health and environmental concerns [1]. Growing evidence of their endocrine-disrupting potential has prompted stringent regulatory action. In the European Union, the REACH (Registration, Evaluation, Authorisation, and Restriction of Chemicals) legislation continues to restrict the use of several phthalates in consumer products [2].

More recently, in 2025, the European Union’s Commission also made additional changes to food-contact material laws, which suggests that the EU will continue to have stricter regulations for these items as well [3]. Similar to this in the U.S., in the latter part of 2025, the Environmental Protection Agency (EPA) proposed regulating five common phthalates because they are believed to be harmful to people [4]. Meanwhile, the Food and Drug Administration (FDA) is still studying the safety of all other FDA-authorized phthalates that are being used as packaging materials [5].

Concurrently, the increasing demand for compostable packaging materials, compliant with standards such as ASTM D6400, has accelerated the transition to safer, biodegradable alternatives [6,7].

Sorbitol esters and other polyol esters particularly have the potential to emerge as the most promising candidates, owing to their high availability, renewability, and safety.

Polyols like sorbitol, as a hydroxyl-rich molecule, can effectively function as a plasticizer. By inserting themselves into the spaces of polymer chains, they increase the distance between polymer chains and thus disrupt the strong, rigidizing intermolecular hydrogen bonding (or other) forces that hold together these hydrophilic polymers. The resultant increase in interstitial space (free volume) provides additional freedom to move polymer chains, leading to increased flexibility and lowering of the material’s glass transition temperature [8,9].

Sorbitol, derived from the hydrogenation of glucose and already used extensively in the food and pharmaceutical industries, has been shown to improve barrier properties and tensile strength when incorporated into biopolymer films [10,11]. Modification of these properties further utilizes the material through the application of chemical modifications: esterification of sorbitol or isosorbide with fatty acids creates low-volatility liquids, and the oligomerization and epoxidation of sorbitol create improved thermal resistance and polarity [8,9].

More recent examples of modified esters include “ECO-SORB”, which provided a sorbitol-based ester that was stable over 200 °C and was rapidly degradable under marine conditions [12].

This represents an important design trade-off. Rapid biodegradation is advantageous in terms of environmental persistence. However, there exists a possibility that susceptibility to hydrolysis may be a disadvantage as far as the potential longevity and/or durability/performance characteristics of the plasticized product in specific real-world application environments where the products will have to endure lengthy exposure to water/moisture [13,14,15].

Recent examples of chemo-enzymatically modifying vegetable oils with sorbitol illustrate that polyol esters can provide high levels of stability during processing as well as environmental stability. An additional major source of aromatic platforms for the design of plastics using sustainable technologies includes cardanol, a derivative of cashew nutshell liquid (CNSL). Cardanol’s phenolic group and unsaturated aliphatic chain allow for the formation of esters, ethers, and epoxides with varied properties [16]. Cardanol esters have phthalate-like properties due to the presence of an aromatic core with a flexible aliphatic tail and provide enhanced mechanical properties and reduced volatility [16]. Additionally, epoxidized cardanol derivatives have two roles in plastics: they act as plasticizers and also stabilize against PVC degradation by scavenging hydrogen chloride [17]. Furthermore, comparative toxicological studies have demonstrated that many cardanol plasticizers are non-endocrine disrupting and therefore capable of being used in sensitive applications such as food-contact or medical-grade uses [18].

Several new review papers and laboratory experiments suggest that cardanol-derived systems may be good substitutes for the current phthalate-based plasticizers [16,17,18,19,20]. To use cardanol-derived systems on a commercial production scale, there is still much work to be done to establish a standard migration performance and also to determine their overall durability over time in a variety of different service environments. The determination of these factors will rely on highly sensitive multi-analyte chromatography techniques that are able to detect very small quantities of additive and co-formulated molecular species within the complex polymeric matrices illustrated by the most recent developments of HPLC analysis for polymer micelles [21].

Limonene, a monoterpene produced from citrus peel waste, represents an example of the use of agro-industrial waste to produce functional materials. The high volatility of neat limonene may be alleviated through chemical modification. Poly limonene oxide, synthesized through ring-opening polymerization, has been found to significantly increase the ductility, thermal stability, and hydrophobicity of PLA at relatively low loadings and remains compostable [22]. Grafting molecularly onto the polymer backbone, i.e., synthesis of limonene-functionalized polyhedral oligomeric silsesquioxane (POSS) cages, has been found to improve processing and mechanical properties in 3D printing [23].

Although limonene and its derivatives have been shown to be a useful alternative for plasticizing polymers, there are also some issues when using them. Limonene’s strong citrus fragrance can lead to an undesirable taste or aroma in foods packaged in plastics made from limonene-based plasticizers since limonene molecules will often remain within the polymer matrix until they migrate into food [24]. Additionally, because limonene contains double bonds (i.e., it has multiple carbon–carbon double bonds), the limonene molecule can oxidize easily. Oxidation can cause changes to the chemical structure of the limonene, affecting the performance of the plasticizer over time [25,26]. Antioxidants can be added to reduce this problem; however, even when antioxidants are present, limonene is still prone to self-oxidation while stored. The oxidation products created by this process can create potential allergens and possibly interfere with both the functionality of the materials used for packaging and/or the safety of the packaged foods.

Linalyl and geranyl acetate monomeric terpenoid esters are effective plasticizers. However, polymerizing their volatility requires reactive extrusion strategies that bind them chemically to the polymer matrices in order to stop them from migrating and save on ductility over time [27]. Furthermore, syntheses of D-limonene together with zinc oxide nanoparticles lead to multi-functional PLA nanocomposites with antimicrobial activity, and these versatile PLA nanocomposites are promising in meeting the challenges for sustainable food packaging [28]. However, translation of laboratory results to industry practice is not complete. Similar comparative studies regarding long-term aging and cyclic stress resistance, as well as environmental degradation, especially under marine and soil ecological conditions, are sparse [12]. Further regulatory harmonization is still insufficient, and further optimization is needed in how to scale the synthesis methods, especially enzymatic or solvent-free procedures [17].

This paper outlines the most recent progress in natural plasticizers. We focus on three types of natural plasticizers (sorbitol/polyol esters, cardanol derivatives, and limonene-based) and describe how different chemical modifications will impact several key performance indicators, including thermal stability, mechanical properties, migration, and biodegradability.

## 2. Sorbitol and Polyol Esters

### 2.1. Raw Materials and Source

Polyol-based plasticizers (such as sorbitol) have several sustainable characteristics and a variety of useful properties in polymers. Since they are made from vegetable oils and plant sugars, they provide an alternative to fossil fuels and decrease greenhouse gases that are emitted by burning fossil fuels [10,12]. Sorbitol, which is produced via hydrogenation of glucose derived from starch, is a common hydrophilic plasticizer [8].

### 2.2. Chemical Structure and Key Features

Sorbitol exhibits significant water solubility and numerous hydroxyl groups that provide it with the substantial ability to improve the mechanical properties of films and coatings, such as tensile strength, while also contributing to effective barriers to oxygen and moisture.

### 2.3. Physicochemical and Mechanical Properties

**Figure 1:**
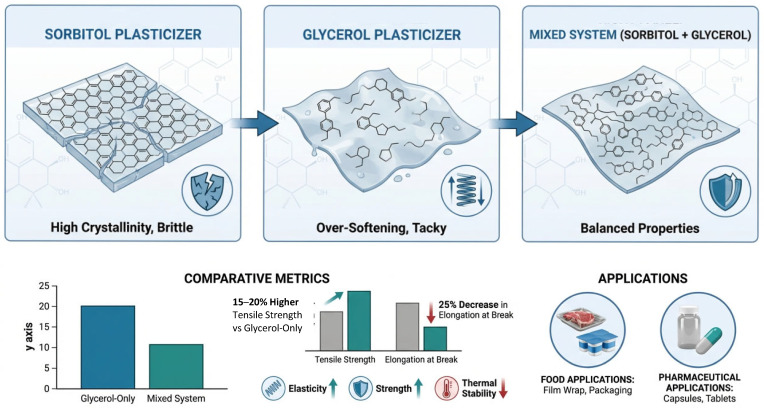
Comparative Analysis of Plasticizer Effects on Biopolymer Film Properties. (**Top**) The infographic illustrates the distinct mechanical impacts of individual and blended plasticizer systems. (**Left**) Sorbitol-only films exhibit increased crystallinity and inherent brittleness. (**Middle**) Glycerol-only formulations lead to over-softening and surface tackiness. (**Bottom**) (**Right**) Mixed sorbitol–glycerol systems (15% *w*/*w*) provide a balanced profile, achieving 15–20% higher tensile strength compared to glycerol-only systems, despite a 25% reduction in elongation at break. The resulting films offer optimized elasticity, strength, and thermal stability, making them ideal for specialized food and pharmaceutical packaging applications. Create by https://scispace.com/.

Substituting sorbitol or polyol ester-based plasticizers for conventional petrochemical plasticizers not only provides a greater degree of sustainability to a polymer system but has been shown to improve flexibility, processability, and functionality. For example, as reported for pullulan films, the addition of sorbitol was found to improve both the strength and barrier properties of the film. On the other hand, glycerol was primarily found to increase the flexibility (elongation) of the films. Although high amounts of sorbitol have been shown to cause brittleness or crystallization, high amounts of glycerol may produce films that become too flexible (soft), sticky, or tacky. It has therefore become common practice among formulators to use blends of these two polyols at a fixed total load. In this context, it has been demonstrated that using equal parts (a 1:1 ratio) of glycerol and sorbitol (at 15 wt%) produces greater tensile strength than films made with either glycerol only or sorbitol only, at the same total load of 30 wt% while maintaining the desired elongation at break as shown in Figure 1 [10].

The desirable properties exhibited by these blended biopolymer films, such as elastic film properties, strong film properties, and thermally stable capsule properties, make them ideal for use in food and drug products. The addition of sorbitol to melt-processed polyvinyl alcohol (PVA) films has also resulted in a significant reduction in the glass transition temperature (Tg) with an increase in the elongation at break and a decrease in the Young’s modulus, thus confirming its high plasticizing efficiency [29].

### 2.4. Chemical Modification Strategies

Chemical modification of polyols or modifying the chemical properties of natural polyol-containing oils has expanded both the application scope and property range of polyols beyond neat polyols. One method to expand this range is through the production of polyol ester-based plasticizers via the reaction of polyols with fatty acids or through modification of vegetable oils [8].

**Figure 2:**
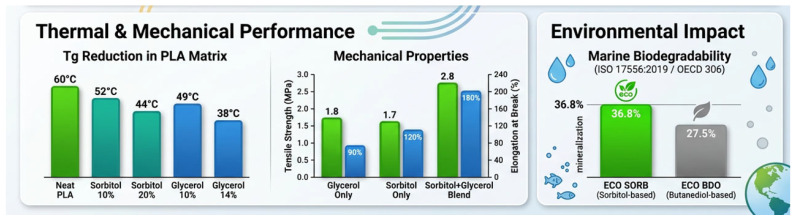
Sorbitol and Glycerol as Polyol-Based Plasticizers: (**Left**) bar chart comparing the glass transition temperature (Tg) reduction in a PLA matrix for sorbitol and glycerol at 10% and 20% loading levels and dual-axis bar chart comparing tensile strength (MPa) and elongation at break (%) for glycerol-only, sorbitol-only, and sorbitol–glycerol 15:15 blend formulations at 30 wt% total plasticizer loading in pullulan films [10]; (**Right**) quantitative comparison of marine biodegradability (% mineralization at 21 days) between ECO-SORB and ECO-BDO, conducted according to ISO 17556:2019 and OECD 306 protocols [12]. Enhanced by https://scispace.com/.

An example of such a procedure done by researchers includes the description of a chemo-enzymatic process starting from cardoon oil. Cardoon oil was initially epoxidized followed by ring opening with alcohol and polyols, including sorbitol, under acidic conditions. The reaction produced a covalent linkage between the polyol and fatty backbone, producing esters with varying degrees of polarity, molecular weights, and compatibility. Sorbitol-modified cardoon oil (ECO-SORB) had a low melting point, remaining a viscous liquid at ambient temperature, providing desirable characteristics for maintaining flexibility under normal operational conditions. Although the addition of sorbitol reduced the onset of thermal degradation relative to its 1,4-butanediol modified counterparts, the material displayed an onset of thermal degradation at 200 °C (TGA analysis using a 10 °C/min heating rate under N2), suitable for standard thermoplastic processing temperatures (180–200 °C), as seen in Figure 2 [12].

Although this research presents an opportunity for the use of ECO-SORB as a plasticizer, there is currently no published information regarding ECO-SORB’s performance as a plasticizer. Therefore, additional study (i.e., the effects of ECO-SORB on the thermal glass transition temperature of a polymer, the tensile modulus of a polymer, etc.) would be required to determine whether it has sufficient performance characteristics that could make it suitable for use in polymers (e.g., PLA).

### 2.5. Environmental and Toxicological Profile

Studies on life-cycle assessments (LCAs) have shown that producing bio-based polyols has several benefits, including less greenhouse gas emissions than their petroleum-based counterparts, because the plant biomass is utilizing CO_2_ for photosynthesis while it is growing [30,31]. Additionally, the bio-based polyols are derived from natural sources and are structurally similar to biological molecules; therefore, they exhibit the inherent characteristics of biodegradability which reduces the likelihood of them persisting in the environment as pollution [8]. The modern eco-design paradigm is to design the plasticizer to be cleavable so that when it reaches the end of its life cycle it can be enzymatically or hydrolytically degraded [12].

Importantly, the incorporation of sorbitol into the ester backbone enhanced biodegradability. The sorbitol–fatty acid ester (ECO-SORB) showed 36.8% mineralization (Dt21) at 21 days when incubated at 21 ± 1 °C with seawater inoculum from the Adriatic Sea (test concentration = 100 mg/L), outperforming the butanediol-based analogue (ECO-BDO, 27.5% mineralization) under identical testing conditions [12], using marine biodegradation tests conducted according to ISO 17556:2019 and OECD 306 guidelines.

Improved degradation of the sorbitol-modified ester was attributed to the higher hydrophilicity and lower crystallinity imparted by the addition of sorbitol, facilitating microbial enzyme accessibility. Such findings have also been reported for other polyol ester plasticizers that contain flexible ester linkages and bio-based functional groups that accelerate breakdown in environmentally relevant conditions [6,8].

### 2.6. Synthesis–Application Insight

Sorbitol and polyol ester-derived plasticizers illustrate how renewable feedstocks, smart molecular designs, and focused chemical manipulations can come together in the formulation of high-performance, low-toxicity additives. Through modification of the molecular architectures based on esterification, epoxidation followed by polyol coupling or enzymatic functionalization, flexibility, thermal resistance, and solubility can be optimized in a manner that does not hinder the biodegradable behavior [8]. This tunable behavior, environmental compatibility, and end-of-life degradability make sorbitol and other polyol ester plasticizers promising candidates for bioplastics and environmentally friendly polymer applications of the future.

This section and the related literature are summarized in Table 1, which compiles representative bio-based polyol and polyol ester plasticizers—including their sources, synthesis routes, physicochemical properties, applications, biocompatibility and toxicity profiles, as well as environmental and economic aspects [32,33,34,35,36,37,38,39,40,41,42]. 

## 3. Glycerol Derivatives

### 3.1. Raw Materials and Source

With their low cost as well as excellent plasticizing efficiency, glycerol-based plasticizers replace many conventional phthalates that persist in ecosystems and pose health hazards [43]. By using glycerol as a renewable by-product, these plasticizers obtained from biodiesel production are a low-cost, abundant source of bio-based feedstock from which safer plasticizers can be derived [44]. This waste valorization not only reduces the dependency on fossil resources but facilitates the circular economy by turning low-value derived waste streams into functional additives.

### 3.2. Chemical Structure and Key Features

Although neat glycerol is very polar and thus a good plasticizer for many hydrophilic materials, it is also extremely small in molecular size and very hydrophilic. These properties may be contributing to the volatility (evaporation), migration, and poor compatibility of glycerol with hydrophobic matrices.

### 3.3. Physicochemical and Mechanical Properties

The addition of 20 wt% of the studied plasticizers lowered the glass transition temperature (Tg) down to 45 °C and increased elongation at break to around 435% from about 6% for pure PLA [45]. However, it should be mentioned that the large extent of improved flexibility is very much dependent on the number of plasticizers used and their compatibility with the PLA [46]. While this means a big increase in ductility, it normally goes along with a decrease in tensile strength and elastic modulus, which are typical characteristics of plasticized polymer systems [47,48].

It has been found that 40 phr of some glycerol-based plasticizers minimized the Tg by 54–86 °C and increased elongation at break to 97% in comparison, with dioctyl terephthalate (DOTP) at the same temperature. For both PLA and PVC, derivatives which had longer alkyl chains showed better miscibility with the polymer [44,45], whereas shorter-chain analogues preferred phase separating, which was also observed using scanning electron microscopy.

Migration was additionally decreased by chemical changes as well. In accelerated leaching studies, glycerol derivatives with either a longer chain length, or one with branch points, migrated to a polar food simulant, typically from 2- to 6-fold lower than those of shorter-string glycerol derivatives [44]. Frequently, these migration phenomena can be modeled utilizing Fick’s Second Law of Diffusion; this law describes how fast the plasticizer is moving through the polymer matrix. Therefore, a smaller diffusion coefficient, which is generally seen when there is a larger molecular weight and/or greater strength of interaction between the polymer and the plasticizer, will result in less migration. Similar patterns have been observed in all water, ethanol (10% *v*/*v*), and acetic acid (3% *v*/*v*) migration testing that has been completed on mixed PVC, and it has also been observed that large and more hydrophobic derivatives exhibit significantly less leaching [45].

In addition to variations in chain length, multi-functional esters, like glycerol trilevulinate (GT), provide application versatility. GT has been demonstrated to produce considerable plasticization in amorphous (PLA and PVC) and semi-crystalline (polyhydroxybutyrate and polycaprolactone) polymers, which will reduce Tg, stiffness, and in some cases even the melting temperature and crystallinity of the resulting polymers [43].

### 3.4. Chemical Modification Strategies

The chemical modification of glycerol allows for the creation of new compounds which are more stable, compatible, and functional.

One approach is the esterification of glycerol with bio-based acids or anhydrides. Glycerol succinate plasticizers have been synthesized by reacting glycerol with succinic anhydride and various alcohols and systematically changing the length of the alkyl chains as a function of the polymer synthesis. Structural changes, especially those that were longer or branched alkyl groups, significantly influenced performance [45].

**Figure 3:**
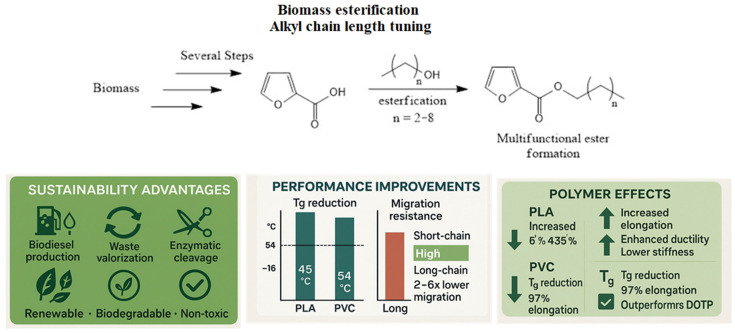
Glycerol-Based Plasticizers: Sustainability, Chemical Modifications, and Performance Enhancement. (**Top**) Esterification strategies with alkyl chain length varying and multi-functional ester formation. Generated by ChemDraw Ultra 12.0.2.1076. (**Bottom**) Performance data: In PLA at 20 wt% loading, glycerol succinate plasticizers reduced Tg by up to 45 °C and increased elongation from 6% (neat PLA) to 435%. In PVC at 40 phr, Tg decreased 54–86 °C with elongation reaching 97%, outperforming DOTP (75% elongation). Longer alkyl chains exhibited 2–6-fold lower migration rates into food simulants (PLA) and 2–4-fold reduction in PVC. GT demonstrated versatility across amorphous and semi-crystalline polymers. Data from [43,44,45]. Generated by https://scispace.com/.

No unreacted hydroxyl groups are part of their tri-ester structure, which enhances their compatibility and plasticizing efficiency yet leads to stability. GT therefore addresses the aforementioned key gap, achieving the same plasticizing efficiency that chain-extended glycerol derivatives achieve while maintaining the enzymatic degradability that longer alkyl chains reduce (Figure 3).

Advanced chemical modifications, such as adjusting alkyl chain length, introducing branching, or forming multi-functional esters, can be designed to achieve exceptional flexibility, low volatility, and minimal migration while preserving their green credentials [43,44,45].

### 3.5. Cost and Industrial Feasibility

Glycerol-based plasticizers remain underutilized in industry because synthesis costs remain a barrier to industrial adoption. Long-chain esters are formulated in a multi-step process with careful control of selectivity, which increases manufacturing cost more than the direct protocols that exist for phthalates [44].

One path to lower synthesis cost is through biocatalytic synthesis which occurs under reduced operating conditions and results in no catalyst residues. The exploration of oligomeric glycerol structures that are suitable for migration resistance but also biodegradable is also under consideration [43].

### 3.6. Environmental and Toxicological Profile

Bio-based plasticizers, such as these, are generally biodegradable and relatively low in toxicity. For instance, GT is hydrolyzed by an enzyme back to glycerol and levulinic acid in an enzyme-catalyzed reaction and becomes a benign substrate that can be re-immersed in natural biochemical cycles [43]. Further confirmation of their safety was given in vitro where GT did not show cytotoxic effects in fibroblast assays [43], and other glycerol-based candidates did not adversely affect mammalian cell viability with prolonged exposure time [44]. Its renewability, biodegradability, and safety contribute to the great advantage for use in food packaging, medical devices, and other sensitive applications for glycerol-based plasticizers [43,44].

Migration studies indicate that although chain-extended glycerol analogues extract more readily in water contact scenarios compared with high-molecular-weight polymeric plasticizers (e.g., for larger time scales) [45], combining renewable glycerol-targeted molecular modifications and sustainable chemistry produces high-performance polymer additives without affecting the environment.

Building on the studies discussed in Section 3, Table 2 summarizes and compares a comprehensive set of glycerol-based and glycerol-derived plasticizers, detailing their production pathways from biodiesel waste valorization, chemical modification strategies, physicochemical properties, polymer applications, biocompatibility profiles, and economic considerations [49,50,51,52,53,54].

## 4. Cardanol-Based Plasticizers

### 4.1. Raw Materials and Source

Cardanol-based plasticizers represent an eco-friendly alternative to phthalates, an agricultural by-product of processed cashew, derived from the cashew nutshell liquid (CNSL). Cardanol is a renewable, non-edible feedstock derived from cashew nutshell liquid (CNSL) from the agricultural by-product of the cashew industry, not competing with food [17,19]. As a significant co-product of cashew processing, global production today reaches hundreds of thousands of tons each year [17]. Cardanol use helps in decreasing dependence on fossil-derived chemicals by valorizing this massive agro-waste stream [19,20].

### 4.2. Chemical Structure and Key Features

Cardanol has an array of potential reaction sites available due to its combination of a phenolic ring and an unsaturated aliphatic chain; this makes it possible to tune the properties of cardanol as a plasticizer by way of chemical modifications [16,17]. As such, chemically modified versions of cardanol can be made that have rigid aromatic cores and flexible aliphatic tails similar to those found in phthalates. Therefore, these types of molecules can replicate the functionality of phthalates [16].

### 4.3. Physicochemical and Mechanical Properties

Esters formed from the reaction of carboxylic acid with the phenol hydroxyl group are another way to make a cardanol ester that has better solubility and less volatility than the original cardanol ester. When you choose an acyl group for your cardanol ester, it makes a big difference in how well they work. For example, when cardanol is made into cardanol acetate (an acetate), it will help to add flexibility and strength to your plastics. On the other hand, if you make cardanol into cardanol oleate (an oleate), it may add some strength but also could be less effective as a plasticizer. That is because longer chains have higher melting points and therefore can be less pliable or flexible [19]. The other major method used to modify the chemical structure of cardanol ester is through the formation of an epoxy on the unsaturated carbon chain. By forming an epoxy functional group, this adds polarity to the molecule which allows for increased mixing with many types of polymers that have polar functional groups (such as PVC). Also, by incorporating epoxide functionality into the molecule, it becomes capable of acting as both a plasticizer and a thermal stabilizer. The epoxide portion of the molecule acts as a scavenger of HCl which is generated upon degradation of PVC [18].

Any structural modifications for a polymer are inherently trade-offs. The incorporation of polar groups into the molecule will enhance compatibility of the polymer as well as the efficiency of plasticization. However, they also introduce increased viscosity and may cause color effects. In addition to its natural yellow color, oxidation of cardanol causes it to darken which limits the possible uses of this material when optical clarity is required [19]. Therefore, the best derivative of cardanol will depend upon the optimization of thermal stability, migration behavior, toxicity profile, and the properties of the melted polymer relative to the performance requirements of the final product [16].

### 4.4. Chemical Modification Strategies

Esterification of the phenolic hydroxyl group with carboxylic acids, for example, is a common strategy to produce cardanol esters with improved compatibility and reduced volatility. Another key strategy is the epoxidation of the unsaturated aliphatic chain.

Other changes to chemical properties, including etherification or hydrogenation, allow additional ways to modify properties. For instance, ethylene glycol ethers derived from cardanol glycidyl ethers may be used to improve mechanical properties. However, the process by which they are synthesized (i.e., using epichlorohydrin) is subject to sustainability concerns [16,20]. The hydrogenation of the aliphatic segment of the molecule enhances both oxidation and color stability, but it may result in decreased flexibility. In contrast, dimerization results in derivatives having a higher molecular weight that exhibit lower levels of migration and volatility but typically also increases viscosity [19]. The various strategies available to utilize cardanol are summarized in Figure 3 and illustrate the potential for using cardanol as a platform for developing high-performance and sustainable plasticizers.

### 4.5. Cost and Industrial Feasibility

These derivatives have potential as alternatives to phthalate-based plasticizers since the availability of cardanol (a by-product of the processing of CNSL) provides an abundance of low-cost and less toxic feedstock. Furthermore, the ability to chemically tailor the plasticizer through chemical modification is not accessible using petroleum-derived feedstocks [16,17,18,19,20].

### 4.6. Environmental and Toxicological Profile

Cardanol-based plasticizers have obvious health and environmental benefits. Phthalates, mainly DEHP and DINP which leach from polymers, are endocrine disruptors and have adverse impacts on humans and wildlife [19]. By comparison, some cardanol-derivative plasticizers are non-toxic and free of endocrine-disrupting activity [18]. For example, in vitro, epoxidized cardanol ester demonstrated no hormone-disrupting profile, unlike DINP [18]. Furthermore, this safety profile renders cardanol derivative products the added benefit of having a low reactivity to different reactants, with applications for food packaging, medical equipment, and children being highly favorable [19]. In addition, bio-based additives conform to sustainable chemistry objectives by facilitating the substitution of toxic products in consumer goods [17].

Compatibility of cardanol with alternative biodegradable/bio-based polymer improves their eco-friendly profile. However, in view of the increasing reliance on polylactic acid and cellulose acetate, there is a critical need for plasticizers which are renewable, non-toxic, and potentially biodegradable [16]. The hydrolysable ester bonds in cardanol esters could allow for a more rapid degradation in the environment compared to phthalate esters, and more aromatic or ether-enriched derivatives are more durable overall [20]. More studies are needed to fully evaluate the biodegradability and eco-toxicity of the biofuel, but preliminary results indicate a promising environmental impact [19]. The cardanol derived by CNSL therefore solves the plasticization dilemma as a hybrid polymer with renewable material and sustainability, but there are issues related to performance and safety.

**Figure 4:**
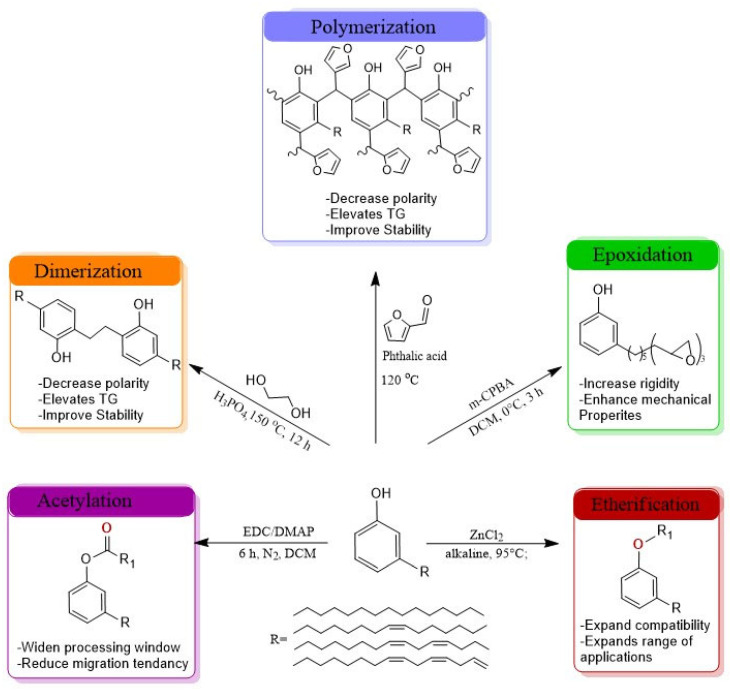
Chemical Modification Methods of Cardanol-Based Plasticizers: Structural Changes and Performance Effects. The central structure represents cardanol while the surrounding panels illustrate key transformation routes, including dimerization [55], polymerization [56], epoxidation [19], acetylation [19], and etherification [20]. Dimerization and polymerization decrease polarity, raise glass transition temperature (Tg), and improve oxidative and color stability. Epoxidation introduces epoxy groups that increase rigidity, enhance mechanical performance, and provide combined plasticizer–stabilizer behavior in PVC. Acetylation and esterification widen the processing window and reduce migration tendency by lowering polarity and volatility, whereas etherification improves compatibility with various polymer matrices and broadens application range. Colored arrows indicate qualitative trade-offs between flexibility, migration resistance, thermal stability, and color stability across the different modification strategies. Generated by ChemDraw Ultra 12.0.2.1076.

The main chemical modification pathways of cardanol relevant to plasticizer design are summarized schematically in Figure 4, highlighting dimerization, polymerization, epoxidation, acetylation, and etherification routes and their qualitative effects on performance, based on the studies reported in [19,20,55,56].

Examples of some of the extraction methods, synthetic modifications, physical attributes, target markets, hazard assessments, life-cycle impact assessments, and economic implications for these materials are summarized in Table 3 [57,58,59,60,61,62].

## 5. Limonene-Based Plasticizers

### 5.1. Raw Materials and Source

Limonene, a cyclic monoterpene recovered from citrus processing waste, provides sustainable alternatives to petrochemical plasticizers [23]. Industrial citrus processing produces over 70,000 tons of limonene annually, a material that can be recovered through distillation with minimal toxic by-products [23]. Utilization of limonene as a polymer additive not only valorizes an otherwise discarded waste stream but supports the adoption of low-toxicity alternatives to petrochemical plasticizers and renewable alternatives.

### 5.2. Chemical Structure and Key Features

Limonene, in its unmodified form, can soften stiff biopolymers (e.g., PLA) and help reduce brittleness. Nonetheless, its relatively low boiling point (~176 °C) overlaps with processing temperature of PLA, thus causing volatilization in melting and transient plasticization [23].

### 5.3. Physicochemical and Mechanical Properties

Poly limonene oxide (PLO) was prepared by catalytic ring-opening polymerization, resulting in a polymeric plasticizer which blends well with PLA. Nevertheless, at just 10 wt%, the flexibility and thermal stability enhancement of PLO was significantly higher, and moisture adsorption was minimized due to its hydrophobic nature [22].

Various limonene moieties were covalently bonded to a silicate cage through hydrolyzations to produce a limonene-derived polyhedral oligomeric silsesquioxane (POSS) modifier. Such a massive and non-volatile “SS-limonene” additive greatly diminished melt viscosity and promoted interlayer adhesion in extrusion-based 3D printing, leading to higher-quality components and reduced printing waste. There was also an enhanced mechanical property that the toughening effect of limonene and the nano-reinforcement from silsesquioxane core contributed to [23].

The incorporation of 10–20 wt% of these plant-derived esters into PLA was shown to significantly improve ductility and increase elongation at break from ~5% to >230%. There was a significant decrement in glass-transition temperature (from ~61 °C to ~40 °C) and a faster crystallization with the respective increase in chain mobility found in the blends [27].

PLA bio-nanocomposites that consist of D-limonene zinc oxide (ZnO) nanoparticles were fabricated, synthesizing films with improved flexibility and antimicrobial activity. Even at 5 wt% ZnO, the films achieved a ~ 99% decrease in *E. coli* strains, but the plasticizing action of D-limonene counteracted the embrittlement due to the inorganic filler [28].

Limonene-loaded electrospun PLA nanofibers were monitored for a period of 12 weeks of storage. They noticed remarkable volatility during the initial two weeks that raised the glass transition temperature and partially refuted plasticization results. However, the antimicrobial effects towards *E. coli*, *S. aureus*, and *B. subtilis* were still exerted for a minimum of a month, suggesting an incomplete retention of limonene within the fiber structure [63,64,65].

### 5.4. Chemical Modification Strategies (If Applicable)

As a result, chemical modification strategies that would retain limonene’s benefits but optimize its compatibility and stability in polymer matrices are as follows:

1. Polymerizing: Limonene polymerized for higher-molecular-weight derivatives with increased stability. Poly limonene oxide (PLO) was prepared by catalytic ring-opening polymerization [22].
2. Covalently bonded: A second good substitute is molecular grafting. A limonene-derived polyhedral oligomeric silsesquioxane (POSS) modifier has been reported in which various limonene moieties were covalently bonded to a silicate cage through hydrosilylation [23].
3. Esterification: Outside of changing limonene by means of itself, terpenoid esters of related nature like linalyl acetate and geranyl acetate have also been considered monomeric plasticizers. Migration behavior is the main drawback of low-molecular-weight plasticizers. To do this, the researchers used reactive extrusion with dicumyl peroxide, which stimulated peroxide-induced grafting from the terpene plasticizers to the PLA backbone. This chemical anchoring decreased leaching and maintained mechanical properties during aging [27].

However, there are many factors that require very close attention when using a peroxide initiator as an additive in reactive extrusion. Overuse of the initiator or excessive temperatures may cause harmful side reactions that could result in degradation of the PLA matrix (chain scission) or cross-linking; either process will severely degrade the mechanical properties of the product.

4. Bio-additives: Limonene has also been utilized as one of the co-added compounds with other bio-additives for such multi-functional materials.

**Figure 5:**
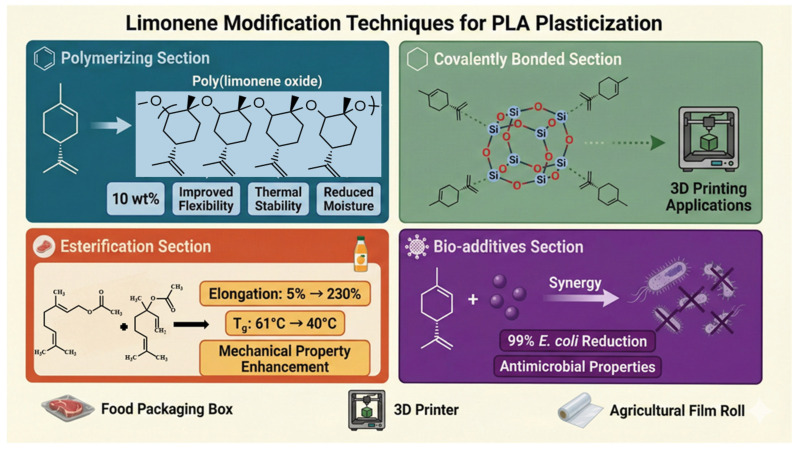
Chemical Modification Methods of Limonene-Based Plasticizers. Structural modifications via esterification, epoxidation, acetylation, etherification, hydrogenation, and dimerization, highlighting effects on compatibility, flexibility, migration, and stability. Generated by https://scispace.com/ structures are revised some redraw by chemDraw.

Methods such as polymeric derivatization, reactive grafting, or encapsulation are recommended to keep the plasticizer working well during the life of the produced product. Volatility is still the principal limit of limonene as an antimicrobial additive with polymers. Its boiling point (~176 °C) is below the thermal processing windows of PLA (200–230 °C), which loses plasticization in the melt compounding process [65]. Three chemical solutions have been developed to tackle that challenge. In a recent study, limonene oxide was polymerized using catalytic ring-opening to produce poly limonene oxide, a non-volatile, plasticizing polymer blend uniform with PLA at 10 wt% [22]. Alternatively, hydrosilylation was utilized to tether limonene with polyhedral oligomeric silsesquioxanes to form thermally stable additives for 3D printing [23]. Reactive extrusion was pursued, employing dicumyl peroxide to covalently graft terpenoid esters to the chains of PLA, achieving a migration reduction of ~60% compared to mixing with a known product [27]. Each has distinct trade-offs. Polymerization is easy to scale but needs catalyst development. Hydrosilylation can be used for new purposes but can be an added headache. Reactive grafting decreases migration but calls for careful processing control. Combined, these changes would make limonene a possible plasticizer for food-contact and agricultural applications where leaching was considered unsuitable for use prior to the present (Figure 5) [22,23,24,25,26,27,28,63].

### 5.5. Environmental and Toxicological Profile

Limonene, a cyclic monoterpene recovered from citrus processing waste, provides a sustainable alternative to petrochemical plasticizers [23]. Utilization of limonene as a polymer additive not only valorizes an otherwise discarded waste stream but supports the adoption of low-toxicity alternatives to petrochemical plasticizers and renewable alternatives. This formulation is completely bio-based or food-contact safe, which is consistent with sustainable packaging practices. Methods such as polymeric derivatization, reactive grafting, or encapsulation are recommended to keep the plasticizer working well during the life of the produced product.

Table 4 summarizes representative limonene-based plasticizer systems, including their feedstock origin, extraction and processing routes, key chemical modification strategies, physicochemical and mechanical properties, polymer applications, and environmental or economic considerations [64,65,66,67,68,69,70].

## 6. Techno-Economic Screening of Bio-Based Plasticizer Routes

This section presents a screening techno-economic assessment (TEA) of potential plasticizers from four different feedstock families. Each assessment assumes 1 kg of plasticizer produced at the plant gate as the functional unit, at a generic multi-ton/year facility producing plastics for 20 years, using a 10 percent discount rate (with neither specific size nor location specified). All assessments utilize metrics of the most significant price (MSP), capital expenditures (CAPEX), operating expenses (OPEX), internal rates of return (IRR), and time to payback based upon recently published studies. The sensitivity of each assessment to varying parameters such as feedstock prices, yield, solvent recovery, and scale is also noted qualitatively (i.e., lower feedstock costs or increased yields will significantly impact MSP/IRR).

### 6.1. Sorbitol Esters

Using sugar-based sorbitol as a feedstock and reporting on the production of sorbitol at an MSP of approximately $0.48-$0.81 USD/kg (produced simultaneously with itaconic acid) with IRRs ranging from 18.5 to 23.6%, we have used the higher estimate ($0.8 USD/kg) as our assumption regarding the cost of sorbitol. Additional capital expenditures to produce sorbitol esters via the chemical modification of sorbitol oligomers will be comparable to those required to construct a fine chemical manufacturing facility. There is currently no publicly disclosed TEA for producing sorbitol esters from sorbitol. Based on the input cost of sorbitol alone and typical yields during hydrogenation, the estimated cost of the resulting plasticizer should increase proportionally with respect to the cost of sorbitol. Cost drivers for both the manufacture and sale of sorbitol esters will primarily depend on the cost of glucose (the source material for sorbitol) and hydrogen; however, energy to separate components will also play a role. Additionally, the economic benefits associated with the production of non-plasticizer co-products may provide additional benefits to the overall economics of production [71].

### 6.2. Glycerol-Derived Systems

For triacetin (glycerol triacetate), a 120 kt/yr plant was simulated in 2025, with glycerol conversion of 96% and triacetin yield of 75%. Based on their simulation results, they reported a CAPEX £ 130.4 M, which equates to about $18 million USD and a break-even MSP of £1754/t (£2.2/kg). At a 10% discount rate, this resulted in an IRR of approximately 16.8% and a payback period of approximately 4.6 years. Energy-intensive distillations (approximately 177 MJ/kg) and glycerol feedstock are primary cost drivers; therefore, control over both glycerol pricing and the energy efficiency of glycerol recovery will be important to reducing costs [72]. For glycerol carbonate (via CO_2_ insertion), an MSP of $0.628/kg was found for their base-case scenario. Including safety-related CAPEX raised MSP to $0.689/kg. Although no IRR/payback information was presented, they assumed greater than 97% yield and utilized market pricing for CO_2_ (about $50/t). Major drivers of cost are CO_2_ capture cost and catalyst reuse through recycling [73].

### 6.3. Cardanol/CNSL Derivatives

Unfortunately, there are no open TEA numbers available for cardanol/CNSL-derived plasticizers. However, due to supply chain limitations, process complexity, and other cost factors, we believe that high multi-million-dollar CAPEX will be necessary for multi-step processes. Therefore, we anticipate plasticizer MSP will likely exceed $1/kg. Main cost drivers are CNSL purification/extraction (batch or SC-CO_2_ processing) and multi-step chemistries (epoxidation and grafting). Lower-cost CNSL options exist when crude CNSL is processed rather than purified. No IRR/payback data have been reported for CNSL-derived plasticizers; however, the value of cardanol (a waste product) and economies of scale in cashew-producing regions will affect feasibility [19].

### 6.4. Limonene Derivatives

Limonene oil extracted from citrus peels has a concentration range of 3–5 wt% limonene oil; thus, limonene extraction costs are relatively high. Techno-economic analyses (TEAs) were used along with simulations for extracting citrus oils using supercritical CO_2_, reporting an orange oil MSP of approximately $148/kg (at 99.5% yield at 50 °C and 74 bar). While no CAPEX/IRR was provided, an earlier biorefinery study demonstrated that recovering limonene in situ could result in IRRs up to ~19% vs. ~16% when purchasing additives (using a 10% discount) [74]. Therefore, energy costs related to limonene extraction and waste management are major cost contributors. For limonene carbonate (reaction of limonene oxide with CO_2_), another study reported CAPEX = $161/t − CO_2_ and OPEX = $153/t − CO_2_ (assuming IL catalyst and 90% CO_2_ conversion). These values translate into approximately $0.14–0.16/kg of carbonate product. Major cost drivers for this route are the IL catalyst and CO_2_ compression/purity requirements. No MSP or IRR was reported [75]. Table 5 summarizes the benchmarked KPIs previously listed.

Plasticizer routes utilizing either sorbitol or glycerol exhibit MSPs ranging from approximately $0.5 to $2.5/kg, while limonene extraction is >$100/kg of oil. Feedstock price, reaction yield, and energy-intensively separating components (distillation and extraction) are common cost drivers among all assessed routes. Competitive payback periods will only occur when low-cost feedstocks and substantial co-product volumes are achieved.

## 7. Challenges and Future Perspectives

Emerging opportunities for bio-based plasticizers derived from polyols and natural materials such as sorbitol, glycerol, cardanol, and limonene will offer multi-faceted approaches to biopolymer development and the circular economy due to convergent developments in these two areas. Studies now suggest a dramatic shift toward the use of bio-functional plasticizers, structurally tailored bio-plasticizers that can replace not only phthalates but other traditional plasticizers as well [76]. For example, designing multi-branched cardanol esters to reduce the plasticizer migration of PLA, while simultaneously improving its intrinsic fragility, demonstrates how molecular architecture can be engineered to balance improvements in mechanical, thermal, and long-term compatibility [76]. CNSL-based epoxides are also reported to have improved performance in PVC systems, and this improvement is thought to result from the interaction between the epoxidized cardanol and the cardanol moieties within the CNSL matrix, resulting in enhanced tensile strength and reduced volatility while maintaining processability [77,78]. Carbonate-based polymers derived from citrus waste (for example, poly limonene carbonate) have emerged as being particularly appealing due to their renewable feedstock origin and their ability to tailor thermomechanical properties via copolymerization with menthane oxides or through grafting onto low-Tg acrylate monomers, thus offering possible solutions to the problem of brittleness in industrial processes [79]. 

An increasing trend exists in the design of products characterized by a growing emphasis on the design of the chemical linkages between the feedstock used to create the desired level of sustainability and minimize waste while preserving the properties of the feedstock used to produce them. These advancements represent a progression of design philosophy beyond simply using feedstocks that are renewable, toward systematically integrating predictable hydrolysable bonds, crystallinity, and degradation kinetics into the design of materials, principles that are founded upon the green chemistry indicators of atom economy and process mass intensity [30,31]. 

There are significant barriers to overcome at the technical, economic, and systemic levels. In particular, the primary challenge has been achieving sufficient scale and cost competitiveness for glycerol and sorbitol that exhibit a high degree of hygroscopicity and volatility, thereby limiting their application under humid and elevated temperature conditions and requiring derivatization (for example, esterification of glycerol into isosorbide diesters or polyglyceryl caprates), which introduces synthetic steps and purification complexities [80,81]. Challenges associated with systems for cardanol include variability in batch composition from agricultural waste streams, requiring stringent standardization protocols and quality-by-design processes that are rarely achieved independent of pharmaceutical processing [82,83]. At the global level, there are limited regulatory frameworks. Although EN 13432 and ASTM D6400 provide indications of the potential for compostability, there exist no universal standards for the verification of bio-based content nor for the determination of the acceptable migration limits of novel plasticizers to food-contact materials, thereby providing manufacturers with little guidance regarding market access [84,85].

Life-cycle assessments have shown an interesting trade-off in bio-based plasticizer systems [86,87]. On the one hand, several formulations of cardanol-polyols show very high end-of-life biodegradability; however, these same materials may be subject to energy-intensive epoxidation and purification processes that could eliminate much of the carbon savings from using the bio-based feedstock [86,87]. Similar to cardanol- and limonene-derived plasticizers which generally will have a lower carbon footprint at the beginning of their life than conventional phthalate plasticizers, there are energy requirements associated with the synthesis and processing of most bio-based plasticizers that can greatly magnify their total environmental burden. A recent study comparing LCA results of polylactide film plasticized by limonene to those plasticized by acetyl tributyl citrate (ATBC), a petroleum-based plasticizer, demonstrated that limonene-plasticized polylactide film has an environmental impact similar to petroleum-based ATBC-plasticized films. Therefore, the environmentally beneficial aspects of limonene’s renewable source do not provide a clear environmental benefit when accounting for the processing costs associated with limonene [88]. Additionally, a more general quantitative analysis of twelve common plasticizers showed that while bio-based plasticizers were less toxic and provided some advantages in terms of carbon footprint reduction (only 7–12%), the disadvantages include increased resource utilization and greenhouse gas emissions [89]; therefore, a comprehensive cradle-to-grave evaluation is critical to truly evaluate sustainability in addition to simply assessing the carbon footprint [88,89]. 

The comprehensive evaluation allows researchers to examine trade-offs across various stages of product development including the production stage, user exposure, and post-user disposal stages [86,87,89]. There have been many advances in developing bio-based plasticizers; however, a few barriers continue to hinder widespread adoption. Additionally, it would be valuable to conduct systematic evaluations of combinations of different types of bio-based plasticizers (e.g., sorbitol-glycerol, polyol-cardanol, polyol-limonene, etc.) to investigate potential synergy effects on mechanical properties, migration resistance, and processability windows [39].

In addition to addressing the technical and economic issues previously mentioned, future research should focus on a variety of other areas that represent new avenues for commercializing products.

### 7.1. Development of Polymeric Plasticizers from Renewable Resources

A particularly promising avenue is the development of polymeric plasticizers derived from renewable resources. These high-molecular-weight plasticizers exhibit inherently low migration and volatility, addressing two of the major limitations of small-molecule additives [80]. The influence of molecular weight and chain length on migration properties has been systematically demonstrated, with larger molecular structures showing significantly reduced leaching [80].

Biodegradable polyether polyols produced using catalytic ring-opening polymerizations are being developed as an emerging class of polymeric plasticizers which are compatible with biodegradable polymers [22,31,43]. One example is poly limonene oxide, synthesized via the catalytic ring-opening polymerization of limonene oxide. It shows considerable potential as a “green” polymeric plasticizer for PLA, by demonstrating a significant lowering of Tg values and retaining its hydrophobic characteristics at loadings as low as 10 wt% [22]. The use of renewable polyol-based biodegradable polyesters represents an entirely different approach to the development of plasticizers, whereby the plasticizer itself is a biodegradable polymer that is perfectly compatible with the host matrix [42]. As such, these polymeric plasticizers provide long-term plasticizing effects without the concerns associated with leaching from small molecule additives and represent a complete paradigm shift in plasticizer design philosophy [37,43].

### 7.2. Exploration of Reactive Plasticizers with Covalent Bonding

Migration in polymers continues to be a significant problem throughout industry due to additives that are used to enhance performance. One method to reduce or eliminate migration is the use of reactive plasticizers. Plasticizers which are attached to the polymer chain via a covalent bond will always remain within the polymer (as opposed to being mobile), thus preventing migration [90]. As a result, reactive plasticizers represent a long-term solution to migration problems; they are chemically bonded into the polymer and therefore cannot migrate out of the polymer [90]. The process of attaching plasticizers to polymeric chains involves reacting them with the polymer during processing such as by using reactive extrusion. By doing so, it eliminates migration of the plasticizer and represents a major advancement in reducing or eliminating plasticizer migration [90]. An example of successful application of reactive extrusion with terpenes was achieved using limonene-derived plasticizers. The results produced polylactide formulations that were highly flexible and possessed increased ductility with significantly less migration than other formulations tested [27]. Additionally, limonene derivatives have also proven to be beneficial reactive modifiers for polylactides. Using covalent modification methods, researchers have been able to create functional polylactide-based products with specific properties [23]. Overall, attaching plasticizers to polymeric chains through a chemical reaction, i.e., covalent bonding, presents a fundamental transformation in how plastics are formulated. It converts the plasticizer from a mobile additive to an integral part of the polymer backbone, effectively eliminating migration routes [90]. As such, research regarding the attachment of plasticizers via covalent bonds has received extensive study and has resulted in both improved plasticization along with minimized migration rates [27,90].

### 7.3. Systematic Long-Term Aging and Durability Studies

Further still, it has been identified that additional study is needed to evaluate the durability and long-term aging of bio-plasticized polymer materials in real-world applications. Evaluation of the performance characteristics of bioplastics over an extended period of time, specifically relating to thermal stabilization as well as material resistance to environmental degradation, is key to understanding both the commercial viability and market acceptance of such products. Recent studies evaluating the evolution of the physical properties of electrospun polylactide fibers incorporating limonene (essential oils) at various times during storage under differing conditions has provided valuable insight regarding the retention and stability of plasticizers within bioplastics [63], while the increased thermal stability of limonene-modified cellulose pulp fiber–polylactide composites via the use of bio-plasticizers demonstrate the possibility of achieving enhanced thermal stability when selecting appropriate bio-based plasticizers [64].

Comprehensive tests to determine a product’s long-term durability should address the mechanisms by which biodegradable polyester is degraded in water (hydrolysis) [13] and how moisture affects both the manufacturing and mechanical properties of biodegradable polyesters [14], as well as how environmental and storage conditions affect plasticized polymers [63,64]. A detailed understanding of marine biodeterioration of bio-based oligoesters and plasticizers will provide significant insight into assessing environment-based product performance [12]. Controlled release tests for bio-based plasticizers in different environments are also necessary to predict a product’s performance over its service life [26].

### 7.4. Development of Standardized Testing Protocols and Regulatory Frameworks

More emphasis is now being placed on the development of standardized test methods that measure the properties of bio-based content, biodegradability, and migration. These are required if we wish to see greater regulatory acceptance and consumer confidence. ASTM D6400-19 does provide a standard for labeling those plastics which are to be composted in aerobic municipal or industrial environments. However, while the European Chemicals Agency has listed certain phthalates as restricted via Annex XVII to REACH [2,6], there remains considerable uncertainty about how best to verify bio-based content and what should represent an acceptable migration limit for newer types of plasticizers [84]. Bio-based plastics policy analysis identifies a number of gaps and opportunities related to the sustainability of food packaging, along with a potential for regulatory convergence [84]. Recent developments at the U.S. Environmental Protection Agency concerning their phthalate Action Plan [91], as well as recent regulatory activities from the FDA concerning phthalates in food packaging and food-contact applications [5] indicate a rapidly changing regulatory environment. The European Commission’s recent Regulation (EU) 2025/351 amended regulations on plastic materials and articles intended to come into contact with food and includes updated guidelines for appropriate practices [3]. The EPA’s statement of intent to regulate numerous uses of five phthalate chemicals [4] represents the international trend toward stricter control over the use of conventional plasticizers and a need for validated replacements. Standardized test methodologies for assessing the performance characteristics of bio-based plasticizers, across national borders, is important because such standardization will allow manufacturers to better understand their options regarding product placement within markets and facilitate the commercialization of new laboratory-scale technologies. A comprehensive textbook on plasticizers provides a foundational understanding of the basis upon which such standardized test methodologies can be developed [1].

An interdisciplinary approach that includes collaboration among chemists, material scientists, policy architects, industrial ecologists, and supply chain planners will be required to transition laboratory-scale promise into production-ready, globally applicable strategies, as described in a systematic review in 2025 [92].

## 8. Conclusions

This review comparatively examines five major classes of bio-plasticizers (sorbitol/polyol esters, glycerol derivatives, cardanol derivatives, and limonene-based systems) on four key dimensions: (1) synthesis route, (2) physical/chemical properties, (3) polymer compatibility, (4) toxicological profile, and (5) environmental performance. A number of important design rules were developed based on comparative analysis. For example, sorbitol/polyol esters are characterized by their good biodegradable profiles and good food-contact safety ratings. Glycerol-derived plasticizers provide flexibility in achieving various property profiles depending upon the specific bio-acid used to perform the esterification reaction. Cardanol derivatives from CNSL (cardanol oil) provide aromatic functional groups which act as dual-function plasticizers/stabilizers. The potential for limonene-based plasticizers in PLA is very high; however, some limitations exist due to the inherent volatile nature of limonene-based derivatives and their elimination/reduction via either polymerization or grafting onto PLA.

There are many opportunities for future advancement in all four classes of bio-based plasticizers. However, prior to becoming commercially viable, there are still a number of critical issues that need to be resolved. One of the most pressing concerns for all four classes of bio-plasticizers is migration, especially for the lower-molecular-weight esters. Another area of concern is the long-term oxidative and thermal stability of the unsaturated derivatives from cardanol and limonene. Although advances are being made in all four classes of bio-plasticizers, there are still many impediments to their widespread acceptance as substitutes for petrochemically sourced plasticizers. These include large-scale economic production of the bio-based feedstocks and/or intermediates, producing consistently repeatable results from one batch to another from raw materials obtained from agricultural waste streams and developing international standards and guidelines to regulate the use of new bio-based plasticizers. The use of advanced computer modeling tools and techniques, including machine learning algorithms that have been utilized in formulating pharmaceutical nanoparticles, will likely enhance significantly the ability to rapidly screen and optimize the structure–property relationships associated with bio-plasticizers and the operating parameters associated with their manufacture [93].

The authors suggest that future research focus on the development of high-molecular-weight polymeric and reactive plasticizers, conducting comprehensive long-term migration and aging studies at realistic temperatures, conducting full life-cycle assessments, and developing an interdisciplinary collaboration among researchers in chemistry, polymer engineering, and regulatory affairs to develop a commercial pathway for transitioning laboratory-based promise into a commercially viable product. A concerted, interdisciplinary effort will be required to translate the laboratory-scale promise of these materials into sustainable, market-ready solutions.

The comparative evaluation in this paper is thus a qualitative assessment based upon the limited availability of comparable data for each of the four types of plasticizers studied. The majority of reported research for sorbitol/polyol and glycerol plasticizers provides significant details about their processability (i.e., the temperature range over which they can be processed), migration behavior and, in certain instances, life-cycle assessments. However, there is an almost complete lack of published data on these same subjects with regard to both cardanol- and limonene-based plasticizers.

## Figures and Tables

**Table 1:** Comparative Overview of Bio-Based Polyol and Polyol Ester Plasticizers: Sources, Synthesis, Properties, Applications, and Sustainability.

Sources	Extraction Method	Chemical Modification	Physicochemical Properties	Uses	Biocompatibility and Toxicity	Environmental Impact	Economic Considerations
Sorbitol (fruits/plants); glycerol (biodiesel by-product)	Commercially available; no extraction in study	None (used neat)	Improves flexibility and elongation by reducing H-bonding; glycerol increases permeability and flexibility	Plasticizers for starch-based biodegradable films	Safe; biodegradable; renewable	Eco-friendly; sustainable alternative to synthetic plasticizers	Low cost; abundant (notably glycerol)
Propylene glycol + fatty acids (bio-based)	Esterification of propylene glycol; epoxidation of unsaturated bonds	Polyol esters and epoxidized esters	Improved tensile strength and elongation; reduced swelling; enhanced barrier properties	Plasticizers for sodium alginate antimicrobial food packaging films	Bio-based; food-contact safe; antimicrobial when combined with extracts	Biodegradable; renewable	Cost-effective; scalable esterification
Isosorbide from glucose/sorbitol	Hydrogenation of glucose → sorbitol → isosorbide; esterification	Isosorbide esters	Thermally stable; biodegradable; hygroscopic	Plasticizers for starch polymers and PLA	Non-toxic; hygroscopicity is a limitation	Renewable; biodegradable	Biomass-based; processing complexity adds cost
Commercial sorbitol	Used as purchased	None (neat sorbitol)	Improves flexibility, elasticity, thickness, morphology of alginate–starch films	Plasticizer for alginate–starch films (packaging, pharma)	Safe; antioxidant activity observed	Eco-friendly; renewable	Low cost; industrially available
Sorbitol/isosorbide from glucose	Glucose hydrogenation; esterification with fatty acids	Fatty acid esters	Lower Tg; improved flexibility; reduced migration for high-MW esters	Plasticizers for PLA, PHB, PHBV, starch, cellulose	Non-toxic; phthalate alternative	Fully bio-based; biodegradable	Low cost from biomass; multi-step processing
Isosorbide from sorbitol	Catalytic dehydration; esterification	Isosorbide esters and oligoesters	Reduced Tg; improved ductility; low migration	Plasticizers for PLA and compostable blends	Non-toxic; food-contact suitable	Renewable; biodegradable	Scalable; catalyst-dependent cost
Commercial sorbitol	Used as purchased	None	Improves flexibility; lowers modulus; increases surface energy	Plasticizer for chitosan-based wound dressings	Non-toxic; promotes cell viability (HaCaT)	Biodegradable; medical-safe	Low cost; scalable
Commercial sorbitol	Used as purchased	None	Enhanced tensile strength, crystalline; reduced moisture uptake	Plasticizer for corn-starch bioplastic films	Safe; biocompatible	Renewable; biodegradable	Low cost; suitable for scale-up
Sorbitol (glucose hydrogenation)	Catalytic hydrogenation (Raney Ni)	None (also intermediate for isosorbide)	Humectant; water-soluble; cooling effect; flexible plasticizer	Plasticizer for bioplastics; food and pharma uses	Non-toxic; laxative effect at high dose	Renewable; biodegradable	Low cost; million-ton industrial scale
Commercial sorbitol	Industrial glucose hydrogenation	None	Lowers Tg; increases ductility and flexibility; humectant effect	Plasticizer for PVA and biopolymer blends	Safe; food and medical compatible	Eco-friendly; renewable	Low cost; widely available
Sorbitol from biomass	Hydrogenation of glucose; esterification	Sorbitol esters	Improved flexibility; reduced Tg; enhanced elongation	Plasticizers for starch, PLA, PHB, cellulose	Non-toxic; food-contact safe	Biodegradable; renewable	Cost-effective; industrially scalable
Sorbitol + cardoon oil	Epoxidation of oil; acid-catalyzed ring opening with sorbitol	Sorbitol-modified epoxidized oil (ECO-SRB)	High OH number; Tg −16 °C; enhanced flexibility	Bio-plasticizer and biolubricant	Biodegradable; marine degradation confirmed	Renewable; circular economy	Cost-effective; added processing steps
Commercial sorbitol (with glycerol blend)	Used as purchased	None	Improved tensile strength, crystalline; reduced moisture uptake	Plasticizer for pullulan films and soft capsules	Safe; pharmaceutical grade	Biodegradable; renewable	Low cost; industrially viable
Sorbitol-based polyesters	Hydrogenation of glucose; melt polycondensation	Condensation with diacids/amino acids	High modulus; tunable degradation; biodegradable elastomers	Tissue engineering scaffolds; biomedical composites	Biocompatible; no toxicity reported	Renewable; biodegradable	Cost-effective feedstock synthesis-dependent cost

**Table 2:** Comparative Overview of Glycerol-Based Plasticizers: Sources, Synthesis Routes, Properties, Applications, and Sustainability Assessment.

Sources	Extraction Method	Chemical Modification	Physicochemical Properties	Uses	Biocompatibility and Toxicity	Environmental Impact	Economic Considerations
By-product of biodiesel production (transesterification)	Chemical or enzymatic transesterification; purification by membrane separation or adsorption	Esterification to mono-, di-, and polyglycerol esters	Viscous, hygroscopic, water-soluble, high boiling point (~290 °C)	Food (humectant, emulsifier), cosmetics (moisturizer), pharmaceuticals (plasticizer, excipient)	GRAS; non-toxic; biocompatible	Biodegradable; eco-friendly	Low cost; abundant biodiesel by-product
Biodiesel production from vegetable oils	Glycerol separated during transesterification; melt blending with additives	Esterification and blending with citric acid, benzoic acid, and sunflower oil	Flexible; enhanced PVC compatibility; improved thermal stability; reduced crystallinity	PVC plasticizer; eco-friendly alternative to DOP	Non-toxic; safer than phthalates	Renewable; biodegradable; waste valorization	Low cost; high-value utilization of biodiesel waste
Renewable glycerol from biodiesel	Solvent-free esterification with succinic anhydride and alcohols	Glycerol–succinate derivatives with variable alkyl chains	Tg reduction in PLA (~44 °C); elongation up to 435%; thermal stability up to 293 °C; low migration	PLA plasticizer for food packaging	Non-toxic for C6 or shorter chains; moderate cytotoxicity for longer chains	Bio-based; biodegradable; low leaching	Renewable; scalable; potentially low cost
Biodiesel by-product from fats and oils	Direct recovery from transesterification process	Used directly or modified via esterification/blending	Trihydroxyl polyol; water-soluble; improves flexibility of biopolymers	Plasticizer for starch and cellulose-based biodegradable plastics	GRAS; non-toxic; biodegradable	Renewable; low environmental footprint	Low cost; industrial-scale availability
Biodiesel-derived glycerol from plant oils	By-product of biodiesel transesterification	Esterification to glycerol and succinate esters	Improves flexibility and thermal properties of PVC; reduces brittleness	Plasticizer for PVC films, medical devices, eco-packaging	Non-toxic; phthalate alternative	Biodegradable; low leachability	Abundant; industrially scalable
Commercial glycerol (renewable, biodiesel origin)	Commercially available; no extraction	None (used as pure glycerol)	Improves flexibility; increases moisture uptake; reduces density	Plasticizer for edible Persian gum films	Safe; edible; GRAS	Biodegradable; renewable	Low cost; readily available
Glycerol (biodiesel by-product) and adipic acid	Esterification synthesis	Hyperbranched glycerol–adipate polyesters	Flexible; biodegradable; compatible with compostable polymers	Plasticizer for PLA, PHB, PBAT green packaging	Non-toxic; food-contact suitable	Renewable; biodegradable; low toxicity	Cost-effective; scalable production
Glycerol (biodiesel) and levulinic acid (cellulose waste)	Solvent-free esterification under mild conditions	Glycerol trilevulinate (GT)	Reduces Tg and Tm; improves flexibility; low volatility and migration	Plasticizer for PVC, PLA, PHB, PHBV, PCL	Cytocompatible (IC50 ~4–6 mg/mL in fibroblasts)	Fully bio-based; biodegradable; waste valorization	Cost-effective; scalable
Biodiesel-derived glycerol	Esterification and alkyl functionalization	Glycerol esters with C3–C7 and branched alkyl chains	PVC Tg reduction (54–86 °C); high elongation; DOTP-like thermal stability	Flexible PVC plasticizers	Low cytotoxicity for short chains; mild toxicity for longer chains	Renewable; biodegradable	Low cost; scalable
Renewable malic acid	Esterification with alcohols followed by acetylation	Acetylated malate tri-esters (AcMAE-Cn)	High thermal stability (up to 336 °C); low migration; improved flexibility	Plasticizers for PVC and PLA packaging films	Bio-based; presumed safe (cytotoxicity not directly evaluated)	Renewable; biodegradable; low leaching	Scalable; potentially low cost
Ethylene glycol and natural fatty acids	Microwave-assisted esterification	Fatty acid esters of ethylene glycol	High flexibility; improved thermal stability; enhanced migration resistance	PVC plasticizers for biomedical and antimicrobial applications	Non-toxic; antimicrobial activity when combined with thiazoles	Biodegradable; safer than phthalates	Energy-efficient; cost-effective synthesis

**Table 3:** Cardanol-Based Plasticizers from CNSL: Extraction, Modification, Properties, Applications, and Sustainability Assessment.

Extraction Method	Chemical Modification	Physicochemical Properties	Uses	Biocompatibility and Toxicity	Environmental Impact	Economic Considerations
Cardanol synthesized by reaction with 3-chloro-2-hydroxypropanoic acid followed by cyclization with acetone/H_2_SO_4_	Formation of cardanol-based dioxolanone for PLA copolymerization	Lowers PLA Tg; reduces crystallinity; increases amorphous phase and flexibility	PLA additive to improve flexibility and reduce brittleness	Bio-based; low toxicity expected; no direct biocompatibility assays reported	Renewable; biodegradable within PLA matrix	Cost-effective due to CNSL abundance
Flash column chromatography (MeCN/H_2_O/AcOH, 80:20:1) with solvent recovery (~82%)	Fractionation and optional epoxidation of triene moieties	Phenolic OH; unsaturated alkenyl chains; confirmed by NMR, GC-MS, HPLC, FTIR	Bio-plasticizers, coatings, polymers, adhesives, antibacterial and flame-retardant systems	No specific assays reported; expected lower toxicity than petrochemical analogues	Renewable; agro-waste valorization; solvent recovery improves sustainability	Cost-effective through CNSL utilization and solvent recycling
Steglich esterification with fatty acids followed by epoxidation (HCOOH/H_2_O_2_)	Esterification and epoxidation of phenolic and alkenyl groups	Lower Tg; enhanced flexibility; thermal and mechanical performance comparable to DINP-PVC	Primary PVC plasticizers (phthalate alternatives)	Low ecotoxicity; no endocrine disruption (YES/YAS); safer than DINP	Fully bio-based; biodegradable; eco-friendly	Cost-effective due to CNSL availability; industrially viable
Thermal decarboxylation of anacardic acid followed by chemical derivatization	Esterification, epoxidation, acetylation, etherification, glycidylation	Reduced Tg; improved thermal stability; higher elongation; phthalate-comparable mechanics	Plasticizers for PVC, PLA, cellulose acetate, and rubber	Several derivatives non-toxic and non-endocrine disrupting	Renewable; biodegradable; safer than petrochemical plasticizers	Economical due to CNSL; some routes use costly reagents
Vacuum-distilled cardanol; hydroxyethylation followed by methacrylation	Conversion to cardanol methacrylate monomer	Adjustable Tg (−35 to 10 °C); increased modulus; higher crosslink density	Bio-based latex coatings and films	No direct toxicity data; expected safer than petrochemical analogues	Renewable; reduced VOCs; high biorenewable carbon index	Cost-effective; suitable for coatings industry
Vacuum distillation followed by chemical functionalization	Esterification, sulfonation, quaternization; coupling with glycols, amines, sugars, epoxides	Amphiphilic behavior; reduced surface tension; biodegradable; antimicrobial activity	Surfactants, detergents, coatings, adhesives, bioplastics, plasticizers	Low toxicity; some derivatives antimicrobial with low cytotoxicity	Renewable; biodegradable; petrochemical replacement	Cost-effective; supports circular economy
Solvent extraction and vacuum distillation	Acetylation and epoxidation of cardanol	Lower Tg; enhanced flexibility; improved thermal and mechanical performance in PVC	PVC plasticizers; epoxy diluents; cellulose acetate additives	Bio-based; non-toxic; safer than phthalates	Renewable; biodegradable; agro-waste valorization	Cost-effective; some applications are higher than phthalates
Thermal decarboxylation and vacuum distillation; multi-step functionalization	Epoxidation, ring-opening, esterification, thiol–ene, Mannich reactions to polyols	High OH values (≤553 mg KOH/g); tunable flexibility; good thermal properties	Plasticizers for PU, polyesters, epoxies; coatings and adhesives	Low toxicity; biodegradable; some flame-retardant derivatives	Renewable; supports green chemistry	Economical feedstock: synthesis complexity may affect scale-up
Extraction with rosin acid derivative and esterification (oxalyl chloride/pyridine)	Cardanol–rosin ester plasticizer	Lower Tg; improved elongation; good miscibility with PVC	Phthalate-free PVC plasticizers	Non-toxic; no migration issues reported	Fully bio-based; biodegradable	Cost-effective from agro-waste; moderate synthesis cost
Friedel–Crafts alkylation (100 °C, p-TSA catalyst)	Polymerized cardanol (alkylated phenolic polymer)	Mw ≈ 4 × 10^4^; Tg −20.7 °C; high elongation and rubber compatibility	Plasticizer/compatibilizer for rubber formulations	Bio-based; low toxicity expected; minimal migration	Renewable; solvent-free synthesis	Cost-effective; scalable rubber applications
Hot oil, solvent, or supercritical CO_2_ extraction; vacuum distillation	Esterification, epoxidation, phosphorylation, etherification	Lower Tg; enhanced thermal stability; high polymer compatibility	Plasticizers for PVC, NR, EPDM, EVA; surfactants and adhesives	Non-toxic; phthalate-free; no endocrine disruption	Renewable; biodegradable	Cost-effective; growing CNSL market
Pyrolysis or supercritical CO_2_ extraction; vacuum distillation	Esterification, epoxidation, Mannich, thiol–ene, polymerization	Thermally stable; hydrophobic; strong mechanical performance; low permeability	Plasticizers, coatings, adhesives, resins, rubbers, composites	Low toxicity expected; some flame-retardant derivatives	Renewable; fossil-free alternative	Cost-effective feedstock: purification may add cost

**Table 4:** Limonene-Based Plasticizers: Extraction, Modification, Properties, Applications, and Sustainability.

Sources	Extraction Method	Chemical Modification	Physicochemical Properties	Uses	Biocompatibility and Toxicity	Environmental Impact	Economic Considerations
Limonene (citrus fruits)	Infusion into cellulose fibers followed by hot pressing with PLA	None (physical encapsulation)	Lower Tg; improved flexibility and elongation; sustained antioxidant activity (>3 days)	Active food and cosmetic packaging; antibacterial applications	GRAS; non-toxic	Renewable; biodegradable; eco-friendly	Low cost from citrus waste; volatility requires encapsulation
Polyethylene glycol sorbitan esters (Tween^®^ 20, 80)	Commercially obtained	None (used as plasticizers)	Reduced Tg (PLA 61 → ~29.5 °C); elongation up to 194%; higher impact resistance	PLA plasticizers for packaging and bioplastics	Non-toxic; widely used in food and pharma	Renewable; biodegradable; phthalate-free	Cost-effective; commercially available
d-Limonene (citrus peel oil)	Steam distillation, solvent, supercritical CO_2_, microwave hydrodistillation	None; nanoencapsulation (emulsions, SLNs, NLCs)	Volatile; low water solubility; bp 176 °C; oxidation-prone	Food packaging, cosmetics, pharma (antioxidant/antimicrobial)	GRAS; nanoform toxicity requires further study	Renewable; biodegradable; citrus waste valorization	Low raw material cost: encapsulation increases cost
Plant terpenes (essential oils)	Commercially sourced; blended into starch/CMC/CMS films	None	Lower Tg; higher flexibility; antimicrobial activity	Medical patches, wound dressings, antibacterial films	Low toxicity; dermatologically safe	Renewable; biodegradable	Cost-effective; volatility requires control
d-Limonene (citrus peel oil)	Steam distillation; solvent casting with chitosan	None	Higher flexibility; lower crystallinity; improved UV/WV barrier; reduced tensile strength at high loadings	Antimicrobial food packaging films	GRAS; non-toxic	Renewable; biodegradable	Low cost: stabilization needed
d-Limonene (citrus essential oils)	Electrospinning into PLA nanofibers	None (encapsulation)	Initial Tg reduction (~18 °C) with gradual increase due to evaporation	Antimicrobial wound dressings	GRAS; safe for medical use	Renewable; biodegradable	Low cost; volatility limits durability
d-Limonene (citrus essential oils)	Vacuum loading into mineral carriers; melt compounding with LDPE	Encapsulation in HNTs, MSNs, ZnONPs, molecular sieves	Lower tensile strength; higher flexibility; improved oxygen barrier	Antimicrobial food packaging films	GRAS; effective against *E. col*i	Renewable; sustainable additive	Low-cost feedstock: encapsulation adds complexity
Limonene (citrus peel oil)	Steam distillation followed by hydrosilylation	Hydrosilylation to silicone-modified limonene (SS-Limonene)	Lower Tg; improved rheology, adhesion, flexibility; higher thermal stability	PLA additives for 3D printing and injection molding	Low toxicity expected; long-term data limited	Renewable; waste-reducing	Cost-effective feedstock: reaction optimization needed
Linalool and geraniol (essential oils)	Commercial sourcing; melt compounding and reactive extrusion	Monomeric plasticizers; peroxide-assisted grafting	Elongation increase (~230–298%); Tg decrease (61.5 → 39.5 °C)	PLA packaging materials	Non-toxic; food-contact safe	Renewable; biodegradable	Cost-effective; REX adds complexity
d-Limonene (citrus peel oil)	Steam distillation; sol–gel ZnO synthesis with oleic acid modification	Oleic acid-modified ZnO; limonene blending	Lower Tg; higher crystallinity; antimicrobial activity	Antimicrobial food packaging and biomedical films	GRAS; ZnO provides sustained antibacterial action	Renewable; biodegradable	ZnO functionalization increases cost
d-Limonene (Calamansi waste)	Steam distillation; GC–MS/FTIR characterization	None	Volatile; antioxidant and antimicrobial activity	Plasticizer candidate; anticancer/pharma research	Cytotoxic to MCF-7 (IC50 7.98 μg/mL); lower toxicity to normal cells	Agro-waste valorization; biodegradable	Low cost; scalable
Limonene oxide (from d-limonene)	Steam distillation; catalytic ring-opening polymerization	ROP to poly limonene oxide (PLO)	PLA Tg decrease (~60 → 41 °C at 10 wt%); improved hydrophobicity	PLA plasticizer for packaging and agricultural films	Bio-based; non-toxic	Renewable; biodegradable	Cost-effective; catalyst optimization needed

**Table 5:** Screening TEA Metrics for Each Bio-Based Plasticizer Route.

Feedstock/Route	MSP (USD/kg)	CAPEX	OPEX	IRR (%)	Payback (y)	Key Cost Drivers
Sorbitol/polyol esters	0.48–0.81	NR	NR	18.5–23.6	NR	Sorbitol/glucose price; H_2_ uptake yield
Triacetin (glycerol)	~2.2	18 M$	NR	16.8	4.6	Glycerol feed cost; distillation energy (177 MJ/kg)
Glycerol carbonate	0.628–0.689	NR	NR	NR	NR	CO_2_ feed/purification; ionic-liquid catalysts
Cardanol/CNSL derivatives	NR	NR	NR	NR	NR	CNSL extraction yield; multi-step reaction costs
Limonene (extraction)	~148 (oil)	NR	148 (oil)	NR	NR	Extraction energy (supercritical CO_2_); peel availability
Limonene carbonate	NR	161 $/tCO_2_	153 $/tCO_2_	NR	NR	CO_2_ compression; IL catalyst cost

NOTE: plant-gate, per 1 kg; NR = not reported.

## Data Availability

No new data were created or analyzed in this study.

## Abbreviations

The following abbreviations are used in this manuscript:

AcMAE	Acetylated Malate Ester
ASTM	American Society for Testing and Materials
CNSL	Cashew Nutshell Liquid
DEHP	Di(2-ethylhexyl) Phthalate
DINP	Diisononyl Phthalate
DOP	Dioctyl Phthalate
DOTP	Dioctyl Terephthalate
ECO-BDO	Butanediol-Modified Epoxidized Cardoon Oil
ECO-SORB	Sorbitol-Modified Epoxidized Cardoon Oil
EPA	Environmental Protection Agency
EU	European Union
FTIR	Fourier-Transform Infrared Spectroscopy
GC-MS	Gas Chromatography–Mass Spectrometry
GRAS	Generally Recognized as Safe
GT	Glycerol Trilevulinate
HNTs	Halloysite Nanotubes
HPLC	High-Performance Liquid Chromatography
ISO	International Organization for Standardization
LDPE	Low-Density Polyethylene
MSNs	Mesoporous Silica Nanoparticles
NMR	Nuclear Magnetic Resonance
OECD	Organisation for Economic Co-operation and Development
PBAT	Poly(butylene adipate-co-terephthalate)
PCL	Polycaprolactone
PHB	Polyhydroxybutyrate
PHBV	Poly(3-hydroxybutyrate-co-3-hydroxyvalerate)
PLA	Poly(lactic acid)
PLO	Poly(limonene oxide)
POSS	Polyhedral Oligomeric Silsesquioxane
PU	Polyurethane
PVA	Poly(vinyl alcohol)
PVC	Poly(vinyl chloride)
REACH	Registration, Evaluation, Authorisation and Restriction of Chemicals
ROP	Ring-Opening Polymerization
SLNs	Solid Lipid Nanoparticles
NLCs	Nanostructured Lipid Carriers
TGA	Thermogravimetric Analysis
Tg	Glass Transition Temperature
Tm	Melting Temperature
ZnO	Zinc Oxide
